# COVID and Camus: Reflections on *The Plague,* collective experience, and qualitative inquiry during a pandemic

**DOI:** 10.1177/1473325020973345

**Published:** 2021-03

**Authors:** Elisabeth Stelson

**Affiliations:** Department of Social and Behavioral Sciences, Harvard University T.H. Chan School of Public Health, Boston, MA, USA

**Keywords:** Fiction, insider/outsider, interpretation, qualitative synthesis, subjectivity, reflexivity

## Abstract

As a social worker and qualitative researcher, I read Albert Camus’s *The Plague* as I lay recovering from COVID-19. The existential novella documents the experience of the citizens of Oran, Algeria during a fictional epidemic, and The Narrator’s documentation is explicitly based on qualitative “data” from participant observation, key informant accounts, and document analysis. Camus’s text forces the reader to reflect on what it means to qualitatively study an issue or an event when the researcher is also affected by it. Just as readers of *The Plague* must ponder the objectives and interpretation of The Narrator who is “closely involved in all that he proposed to narrate,” qualitative researchers must contemplate their own assumptions, aims, and subjectivity, which is both foundational and often overlooked in qualitative inquiry. While this is particularly critical when studying shared or collective experiences, like that of a pandemic, these assumptions and aims should always be made transparent in qualitative research. To this end, I suggest a series of reflective questions for researchers to iteratively grapple with throughout the research process.

On March 26th, 2020, I lay propped up by a mound of pillows in bed, alternating between reading Albert Camus’s *The Plague* (*La Peste*) and closing my eyes, too tired and too pained for much else. Just a few days earlier, I started my day like so many others learning to acclimate to COVID-19 stay-at-home-orders. Breakfast, presentable top and comfortable bottoms, and a schedule of back-to-back online conference calls and an onslaught of emails providing updates on the rapidly changing pandemic landscape. As I neared the end of the workday, I paused to realized how suddenly tired I felt. Achy and fuzzy-headed, too. I crawled into bed, took my temperature (low-grade fever), and went to sleep. Like many individuals around the world, my first symptoms of COVID-19 felt more like a mild cold rather than a disease that could so thoroughly disrupt and convulse nations across the globe. A few days later, I experienced noticeable difficulty breathing, which led me to seek hospital care. A heavy, bone-aching constriction of my chest soon followed along with a fatigue that refused to lift even months later.

Quarantined in my apartment, cared for by a partner who was themselves struggling to recover from COVID-19, I felt my frustration in my immune system and dismay in my government rise. These feelings were often punctuated by an almost third-person fascination and curiosity of how my body was conscripted into the most significant public health crisis in a hundred years, which replicated and illuminated centuries of social and health disparities (Bassett et al., 2020; Kreiger, 2020). It is with this personal and collective experiential backdrop that I turned to Camus. Suffice it to say, existentialism is not always my preferred genre during a time of so much unknown, and I cannot say it helped my anxiety much to read about the fictionalized account of an Algerian city beset with bubonic plague. But something seemed to be leading me to look to literature at a time when scientists, government officials, and the media could provide few answers.

Published in 1947, *The Plague* is a short novel depicting the fictionalized epidemic in and subsequent quarantine of Oran, a city on the Algerian Coast, during its period of French colonization (Camus, 1987). The novel is told from the perspective of The Narrator, who withholds their identity until the last chapter with the claim that it will help the reader understand the text they are reading to be an objective documentation of Oran and its citizens during quarantine. The Narrator writes how reports of bubonic plague—a disease previously believed to be impossible to contract in Oran in the 20th century—was dismissed by government officials as it spread rapidly through the city’s neighborhoods. The Narrator demonstrates how the epidemic lead some citizens to find meaning in combating the disease or even benefit from the disruption it caused while others despaired as the numbers of deaths rose and separation from loved ones outside of Oran continued for months. While the text was originally written as an allegory for the German occupation of France during World War II, I was surprised to find that it prompted my own reflection on what it means to document and analyze an experience in which you are professionally invested and by which you are personally affected.

In the first few yellowed and brittle pages of my book, Camus describes how he intended to document the city’s experience, writing, “In any case the narrator (whose identity will be made known in due course) would have little claim to competence for a task like this, had not chance put him in the way of gathering much information, and had he not been, by the force of things, closely involved in all that he proposed to narrate. This is his justification for playing the part of an historian. Naturally an historian, even an amateur, always has *data*, personal or at second hand, to guide him” (Camus, 1987: 7-8). The Narrator then describes the three types of data that he will employ to help the reader understand how the epidemic affected the city and its residents: 1) participant observations, 2) secondary accounts from eye-witnesses, and 3) written document (Camus, 1987: 8). In doing so, The Narrator neatly identifies the three primary types of data most often employed by qualitative researchers.

As I lay recovering in bed reading the book, I gradually lost sight of the fact that The Narrator—as proclaimed in the opening chapter—was some unknown person that was “closely involved in all that he proposed to narrate.” I had forgotten to consider and reflect on The Narrator’s personal and academic aims, objectives, and experiences, despite knowing that this always influences the research process. In the last few pages of the novel, The Narrator disclosed that this “impartial observer” was in fact the primary character in the book, Dr. Bernard Rieux, who was the first to sound warning of the disease, was dismissed by authorities, and subsequently led the charge to care for the dying citizens of Oran. The reader must pause to reflect on this professed “impartiality” when Dr. Rieux writes that he was “summoned to give evidence [i.e. the book] regarding what was a sort of crime, [and] he has exercised the restraint that behooves a conscientious witness. Following the dictates of his heart, he has deliberately taken the victims’ side and tried to share with his fellow-citizens the only certitudes they had in common—love, exile, and suffering” (Camus, 1987: 246). But how can Dr. Rieux be both “impartial” and follow “the dictates of his heart?” How can he be sure that the “certitudes” of “love, exile, and suffering” were actually commonalities among his “fellow citizens?” How might I have read and interpreted the text had the narrator disclosed his true identity at the beginning? Would I have discounted his “data” because of his active participation in the crisis or trusted it more for the very same reason?

Researchers are often driven to study the issues that they personally care about since it gives meaning and purpose to our work and likely makes us more effective at it. However, we often do not research what we are simultaneously experiencing since our disciplines conventionally expect us to be an objective observer (however elusive a goal) and therefore assume this is an inherent liability (King et al., 1994; Miller and Crabtree, 1999a). Trying to qualitatively study the seismic shifts produced by COVID-19—shifts that may impact the researcher’s own mental and physical wellbeing, social relationships, and livelihood—makes this goal even more elusive. This same difficulty is pronounced in *The Plague*, for the reader is left wondering how they should read, interpret, and trust Dr. Rieux’s analysis when he was so personally and professionally immersed in the epidemic himself. Surely the answer is not to avoid embarking on the analysis of an important and timely research question altogether. For to only research phenomena we presume to be wholly separate from our lives can lead to the “othering” of the people, communities, and events being studied. This echoes darkly of the origins of qualitative research in turn-of-the-century anthropological support of racial subordination and the efforts of these researchers to assign as much distance and difference between themselves as members of White “civilization” and their study subjects (Fredrickson, 2002; Mills, 1997).

Our strength as qualitative social worker researchers is to identify inequities in our communities and give voice to these inequities as “conscientious witnesses” (Camus, 1987: 246). If we are to conduct research relating to the inequities exposed by the current pandemic, how then do we intentionally separate our own experiences and perceptions from those of others when so much is shared and currently being emotionally processed by the researcher? For this simultaneous processing can create substantial “blind spots,” further obfuscating the inequities we hope to make visible. This very problem arises in *The Plague.* Whether a true “blind spot” or a literary one, Camus/The Narrator never mentions the impact of the epidemic on non-French Algerian communities—a glaring and ironic omission of the experiences of a colonized people considering it was written as an allegorical story of the occupation of the authors’ own country (Camus, 1987).

The simple and pat answer to these questions raised in this reflection is that our assumptions and theoretical orientation must be made transparent in the work we do (Strauss and Corbin, 1998), something that Dr. Rieux neglects to do at the start of *The Plague*. This statement leads us back to the continual issue of subjectivity, reflexivity, and positionality that nearly always surfaces in discussions of qualitative research (Malterud, 2001; Miller and Crabtree, 1999b; Peshkin, 1988; Ratner, 2002; Roulston and Shelton, 2015). The goal of course is not to detach the researcher from the research process, for as ethnographer Alan Peshkin wrote, “subjectivity is a garment that cannot be removed” (Peshkin, 1988: 17). Rather the goal is to manage our theoretical orientation and assumptions to make the best choice for how to distill meaning from the data (Borkan, 1999; Peshkin, 1988; Roulston and Shelton, 2015).

However, our theoretical orientation and assumptions are noticeably harder to identify and extricate when experiences that we research are shared or collective and unfolding day-to-day, such as during a pandemic. It is all too easy to let the assumption that a shared exposure to an experience yields the same interpretation, outcome, and response in other people’s lives (King et al., 1994). And it is that much harder to identify our own orientations when we are simultaneously trying to reorient ourselves in a rapidly changing world. Still, just like the COVID-19 pandemic is a visceral reminder of the social and health inequalities already permeating our communities, so too might it remind us that there is always some type of shared experience between the researcher and the participant, which can function both as a tool to open conversation through rapport-building and an impediment to understanding the experiences of other people and communities due to unacknowledged assumptions. In fact, research during a pandemic may force qualitative researchers to more overtly grapple with these foundational, omnipresent, and critical issues in qualitative research.

To this end, I suggest the following reflective questions listed in Table 1 to overtly grapple with subjectivity, reflexivity, and positionality while conducting research during a collective experience, such as the COVID-19 Pandemic. These questions are not a one-and-done, but rather should be revisited in an iterative process. Moreover, I suggest that all members of the research team record their responses and share them with one another as is appropriate to generate avenues of investigation and interpretation. Additionally, this might serve as a tool to check the unguarded impact of subjectivity throughout all stages of the research process. I further suggest that this reflective process be made transparent during dissemination of the research to allow the reader to understand how the researcher’s orientation, aims, and experiences were managed and applied during the study. It is only through this overt and transparent grappling that we can cast off the guise of Dr. Rieux’s impossible “impartiality,” remain as “conscientious witnesses,” and portray the trustworthiness of our analyses during times of upheaval and collective experience.

**Table 1.** Reflective questions for researchers when conducting qualitative research with researcher-participant collective experience.

Research phase	Reflective question
Iteratively during study design and data collection	• What about this event/experience makes it collective or shared? What parts are not shared?• How has this collective event/ experience affected my life? What changes has it produced? • How do I feel about these effects and changes? • How do I think this event/ experience affects participants in the study? • How do I think participants feel about these effects and changes? • How does this event/ experience relate to the research study? If it does, how do I want to assess if my assumptions about participants’ experiences are true? • What might participants assume about the impact of this collective experience on me? How might this affect what they say, not say, and do?
Iteratively before and during analysis	• How has my experience/ interpretation of this collective event changed since the data was collected? How might this impact my analytic choices? • If the collective event had affected me in a different way, such as _____, how might this impact my analytic choices? • How might other shared events/ experiences that occur after data collection impact my analytic choices? • What were my assumptions about the research topic and participants prior to and during data collection? How did they evolve or change? How did I end up addressing these assumptions during data collection? • Do I think my earlier assumptions hold? How would I prove *and* disprove these assumptions?
